# NOX1 to NOX2 switch deactivates AMPK and induces invasive phenotype in colon cancer cells through overexpression of MMP-7

**DOI:** 10.1186/s12943-015-0379-0

**Published:** 2015-06-27

**Authors:** Suhrid Banskota, Sushil C. Regmi, Jung-Ae Kim

**Affiliations:** College of Pharmacy, Yeungnam University, Gyeongsan, 712-749 South Korea

**Keywords:** Colon cancer, Invasive phenotype, NOX1, NOX2, AMPK, MMP-7

## Abstract

**Background:**

Although matrix metalloproteinase (MMP)-7 expression is correlated with increased metastatic potential in human colon cancer cells, the underlying molecular mechanism of invasive phenotype remains unknown. In the current study, we investigated the regulatory effects of membrane NADPH oxidase (NOX) and AMP activated protein kinase (AMPK) on MMP-7 expression and invasive phenotype change in colon cancer cells.

**Methods:**

Production of superoxide anion was measured by lucigenin chemiluminescence assay using whole cells and protein extracts (NADPH oxidase activity), and intracellular reactive oxygen species (ROS) by fluorescence microscopy using 2’,7’-dichlorofluorescein diacetate (DCF-DA). Quantitative real-time polymerase chain reaction (qRT-PCR) and Western blotting were used to measure mRNA and protein levels, respectively. siRNA transfection was used to assess involvement of genes in cancer invasion, which were identified by Matrigel transwell invasion assay. Luciferase reporter assay was performed to identify transcription factors linked to gene expression.

**Results:**

Under basal conditions, less invasive human colon cancer cells (HT29 and Caco-2) showed low MMP-7 expression but high NOX1 expression and AMPK phosphorylation. Treatment of HT29 and Caco-2 cells with 12-*O*-tetradecanoylphorbol-13-acetate (TPA) induced an invasive phenotype response along with corresponding increases in ROS production and NOX2 and MMP-7 expression as well as reduced AMPK phosphorylation, which resemble basal conditions of highly invasive human colon cancer cells (SW620 and HCT116). In addition, inverse regulation between AMPK phosphorylation and NOX2 and MMP-7 expression was observed in HT29 cells treated with different concentrations of exogenous hydrogen peroxide. TPA-induced invasive phenotype in HT29 cells was abolished by treatment with Vit. E, DPI, apocynin, and NOX2 siRNA but not NOX1 siRNA, indicating NOX2-derived ROS production induced an invasive phenotype. TPA-induced induction of MMP-7 expression was suppressed by AP-1, NF-κB, and MAPK (ERK, p38, and JNK) inhibitors, whereas TPA-induced expression of NOX2 and its regulators, p47phox and p67phox, was blocked by p38 and NF-κB inhibitors.

**Conclusions:**

Molecular switch from NOX1 to NOX2 in colon cancer cells induces ROS production and subsequently enhances MMP-7 expression by deactivating AMPK, which otherwise inhibits stimulus-induced autoregulation of ROS and NOX2 gene expression.

**Electronic supplementary material:**

The online version of this article (doi:10.1186/s12943-015-0379-0) contains supplementary material, which is available to authorized users.

## Background

Colorectal cancer is the third most common cancer worldwide [1], and metastasis is the major cause of cancer mortality. Small invasive cells at the advancing edge of tumors are recognized as an independent prognostic factor for colorectal cancer [2]. Increasing evidence has established a link between overexpression of matrix metalloproteinases (MMPs), which play critical roles in cancer cell invasion and metastasis by degrading the extracellular matrix, and cancer stage and/or prognosis [3]. In the case of colorectal carcinomas, MMP-7 overexpression is correlated with increased invasion and metastasis. Therefore, MMP-7 overexpression is recognized as an independent marker of colorectal cancer progression [4–10].

Regulation of human MMP-7 gene expression is dependent on activator protein 1 (AP-1), which is a transcription factor that binds to the promoter region of MMP-7 [11–13]. In addition, MMP-7 expression via JNK/AP-1 pathway activation has been recently observed in cultured cells treated with exogenous H_2_O_2_ [14, 15]. Compared to normal cells, cancer cells produce large amounts of reactive oxygen species (ROS) [16], which are involved in multiple signaling cascades related to carcinogenesis, including invasion and migration of cancer cells [17–20]. During the transition to a malignant phenotype, ROS are known to activate mitogen-activated protein kinases (MAPKs), including the ERK, JNK, and p38 pathways, leading to expression of MMPs for tumor invasion and metastasis [21–23]. ROS are produced not only as byproducts of energy metabolism in mitochondria and the cytosol but also as signaling messengers upon activation of various cell membrane receptors for growth factors [24, 25], cytokines [26], and integrin [27] via membrane NADPH oxidase (NOX). Alteration of MMP expression by NOX1-generated ROS has been studied in various types of cells, including K-Ras-transformed normal rat kidney cells [28], human pancreatic cells [29], and prostate cancer cells [30]. However, there has been no such study performed using colon cancer cells.

The microenvironment of tumors is often characterized by an insufficient amount of nutrients available for rapid cell proliferation, leading to alteration of the AMP/ATP ratio and activation of AMP-activated protein kinase (AMPK), an energy-sensing kinase that regulates cell metabolism. Similarly, energetic stress during cancer metastasis is known to activate AMPK [31–33]. Although AMPK has also been shown to be sensitive to oxidative stress [34], its involvement in cancer metastasis is somewhat dependent on the type of stimulus and oxidative conditions. Lysophosphatidic acid-induced activation of AMPK enhances ovarian cancer cell migration [35], whereas exogenous chemical-induced activation of AMPK inhibits melanoma and colon cancer cell migration [36–38]. Increased AMPK activity has been observed in colon cancer cells in response to exogenous H_2_O_2_ [39]. However, it is unclear whether or not ROS-associated AMPK activation is related to NOX1, which is highly expressed in colon cancer cells compared to normal adjacent tissues [40]. On the other hand, AMPK has been reported to suppress phorbol ester-induced ROS production [41], and AMPK deletion is associated with increased NOX activity [42, 43]. Therefore, the precise regulatory relationship between AMPK and NOX needs to be clearly determined in order to develop a proper strategy to block cancer invasion and metastasis.

In the current study, we investigated the regulatory roles of NOX in MMP-7 expression and AMPK activity by comparing invasive behaviors of HT29 and SW620 colon cancer cells. To induce invasion, cells were treated with 12-*O*-tetradecanoylphorbol-1,3-acetate (TPA), which has been demonstrated to induce migration and invasion in several human cancer cells via induction of MMPs [44–46].

## Results

### MMP-7 expression is correlated with invasive phenotype in TPA-treated HT29 colon cancer cells

**Fig. 1:**
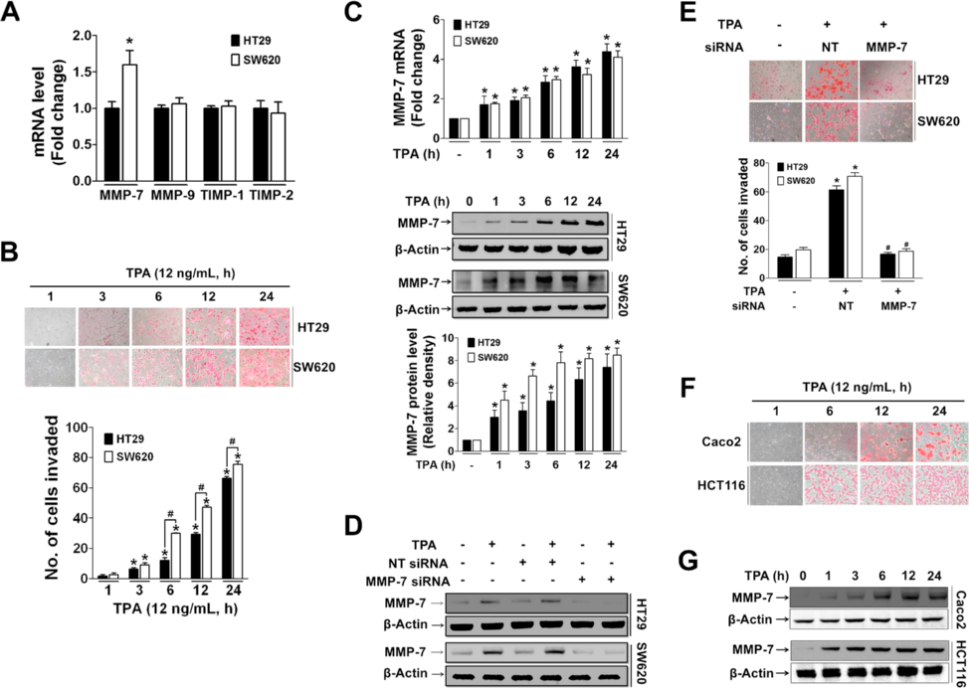
TPA-induced increase in MMP-7 expression corresponds to invasive ability of colorectal cancer cells. **a** Total RNAs from untreated basal HT29 and SW620 cells were analyzed for MMPs by qRT-PCR. The bar graph represents relative expression density of each MMP, and data values are the mean ± SEM from three independent experiments. *P < 0.05 compared to HT29 cells. **b** HT29 and SW620 cells seeded in a Matrigel-coated transwell were treated with TPA in serum-free media, and invaded cells were counted. The bar graphs represent the relative numbers of invaded cells. *P < 0.05 compared to vehicle-treated control group. ^#^ *P* < 0.05 compared to TPA-treated HT29 cells. **c** HT29 and SW620 cells were treated with TPA (12 ng/ml) for the indicated time, and extracted mRNAs and proteins were analyzed for induction of MMP-7 expression by qRT-PCR and immunoblotting. *p < 0.05 compared to vehicle-treated control group of each cell line. **d** Efficiencies of MMP-7 siRNA as measured by Western blotting in HT29 and SW620 cells. **e** In both HT29 and SW620 cells transfected with non-target (NT) and MMP-7 siRNAs, TPA-induced invasion was measured as described in (**b**). *P < 0.05 compared to vehicle-treated control (Mock) group. ^#^ *P* < 0.05 compared to NT siRNA-treated cells. All measurements were performed by three independent experiments. **f, g** TPA induced invasive phenotype change (**f**) in along with enhanced expression of MMP-7 (**g**) in Caco-2 and HCT116 cells

To examine molecular changes, such as MMP expression, in invasive colon cancer cells, less metastatic HT29 cells and highly metastatic SW620 cells were compared. Basal expression pattern of MMPs was similar in both HT29 and SW620 cells, except for a much higher level of MMP-7 expression in SW620 cells (Fig. 1a). Treatment with TPA (tumor promoter) significantly induced invasion of both HT29 and SW620 cells with a more dramatic effect in SW620 cells (Fig. 1b). Invasion of the cells also corresponded to the induction level of MMP-7 (Fig. 1c). In addition, silencing of MMP-7 expression with siRNA (Fig. 1d) completely blocked TPA-induced invasion of both HT29 and SW620 cells (Fig. 1e). Similar changes in invasion (Fig. 1f) and MMP-7 expression (Fig. 1g and Additional file 1: Figrue S1) were also observed in TPA-treated Caco-2, a well-differentiated and non-invasive colon cancer cell line, and HCT116, another invasive colon cancer cell line.

### Molecular switch from NOX1 to NOX2 combined with NOX2-derived ROS production induces expression of MMP-7 and invasion of TPA-treated colon cancer cells

**Fig. 2:**
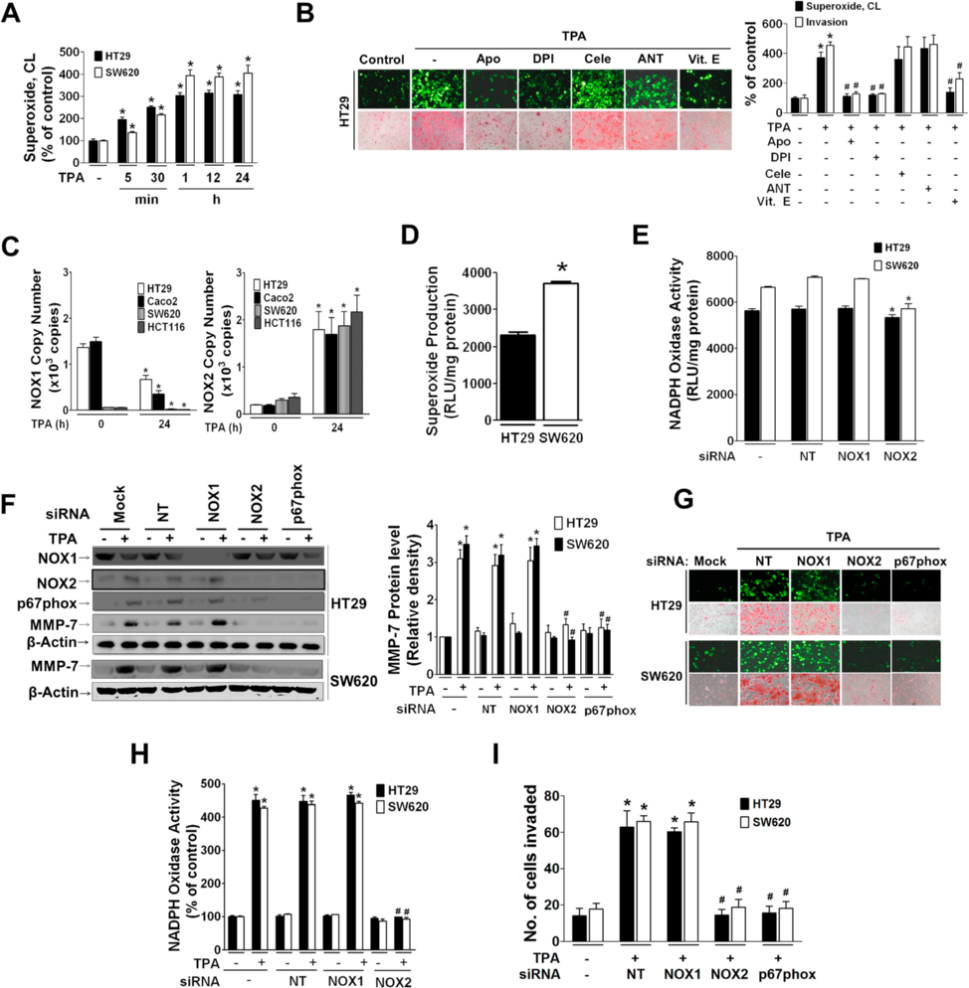
NOX-2 derived ROS enhances MMP-7 expression and invasion of TPA-treated colon cancer cells. **a** Superoxide production in TPA (12 ng/ml)-treated HT29 and SW620 cells was measured by chemiluminescence (CL) using lucigenin (400 μM). **b** HT29 cells were pretreated with Apo (100 μM), DPI (10 μM), Cele (20 μM), ANT (10 μM), or Vit. E (50 μg/mL) for 1 h prior to TPA treatment for 1 h. Intracellular ROS level using DCF-DA (upper panel) and invasion (lower panel) were determined by fluorescence microscopy and Matrigel invasion assay, respectively. The bar graph represents quantitative data on ROS as measured by lucigenin chemiluminescence assay and invasion. Apo, apocynin; DPI, diphenyleneiodonium; Cele, celecoxib; ANT, antimycin A. *P < 0.05 compared to control group. ^#^ *P* < 0.05 compared to TPA-treated cells. **c** Basal mRNA expression of NADPH oxidase components was measured by qRT-PCR in HT-29 and SW620 cells. The data represents mean ± SEM from three independent experiments. *P < 0.05 compared to HT29 cells. **d** Basal superoxide production normalized by cellular protein content was compared between HT29 and SW620 cells. *P < 0.05 compared to HT29 cells. **e-h** Cells were transfected with siRNA specific to NT, NOX1, NOX2, or p67phox. Basal NADPH oxidase activity (**e**) was measured by using lucigenin chemiluminescence. *P < 0.05 compared to NT-treated group. TPA-induced expression of NOX1, NOX2, and MMP-7 (**f**), production of ROS (**g**, upper panel of each cell line), and invasion (**g**, lower panel of each cell line) were examined in both HT29 and SW620 cells. The bar graph represents the NADPH oxidase activity measured by lucigenin chemiluminescence using protein extracts after transfection with siRNA (**h**) and number of invaded cells (**i**). *P < 0.05 compared to vehicle-treated control group. ^#^ *P* < 0.05 compared to TPA-treated cells

High expression of NOX1 is observed during colon cancer, and ROS act as signaling molecules in migration and invasion of cancer cells [15, 44]. Therefore, we next examined whether or not TPA induces NOX-derived production of ROS as well as invasive cell behavior. TPA increased ROS production in both HT29 and SW620 cells in a time-dependent manner, reaching a maximum level at 1 h that was maintained until 24 h (Fig. 2a). In HT29 cells, TPA-induced ROS production was suppressed by pretreatment with DPI and apocynin (NADPH oxidase inhibitors) as well as antioxidant vitamin E (Vit. E) but not celecoxib (COX-2 inhibitor) or antimycin A (mitochondria inhibitor). Similarly, TPA-induced Matrigel invasion was suppressed by DPI and Vit. E but not celecoxib or antimycin A (Fig. 2b). These results indicate a significant role for NADPH oxidase-derived ROS production in invasion of HT29 cells.

We next examined which NOX isoform is responsible for ROS production in colon cancer cells, which reportedly express a high level of NOX1 and moderate level of NOX2 [40, 47]. NOX isoforms were differentially expressed between less metastatic (HT29 and Caco2) and highly metastatic (SW620 and HCT116) colon cancer cells, as shown in copy number measurement of NOX1 and NOX2 mRNA (Fig. 2c). In HT29 and Caco2 cells, the basal level of NOX1 was highly expressed, and treatment of the cells with TPA reduced the NOX1 mRNA copy number, whereas low level of basal NOX2 expression in the cells was dramatically increased by TPA treatment. In SW620 and HCT116 cells, basal NOX1 expression was trace level, which was further decreased by TPA treatment, whereas basal NOX2 expression in SW620 and HCT116 cells was relatively higher than that in HT29 and Caco2 cells, and TPA treatment dramatically increased NOX2 mRNA copy number. The two cell lines were different in producing ROS at the basal level without TPA, higher ROS generation in SW620 cells than in HT29 cells (Fig. 2d). To determine which NOX isoform participates in TPA-induced ROS production and HT29 cell invasion, we performed siRNA transfection. In both HT29 and SW620 cells, silencing of NOX2 but not NOX1 inhibited basal NADPH oxidase activity (Fig. 2e). Moreover, knockdown of genes using siRNAs specific to NOX2 and its activator p67phox but not NOX1 significantly suppressed TPA-induced expression of MMP-7 in HT29 cells as well as in SW620 cells (Fig. 2f). In addition, even though NOX1 was highly expressed in HT29 cells, silencing of NOX1 did not inhibit TPA-induced ROS production, as measured by fluorescent microscopy using DCF to detect intracellular H_2_O_2_ (Fig. 2g) and NADPH oxidase activity to detect superoxide anion (Fig. 2h). This result was also observed in SW620 cells. However, siRNAs specific to NOX2 and p67phox significantly blocked TPA-induced ROS production as well as cell invasion (Fig. 2g). Further, TPA-induced invasion was significantly suppressed by NOX2 and p67phox siRNAs but not NOX1 siRNA in both HT29 and SW620 cells (Fig. 2i).

### NOX2-derived ROS regulates NOX1, NOX2, and MMP-7 expression through the MAPK pathway

**Fig. 3:**
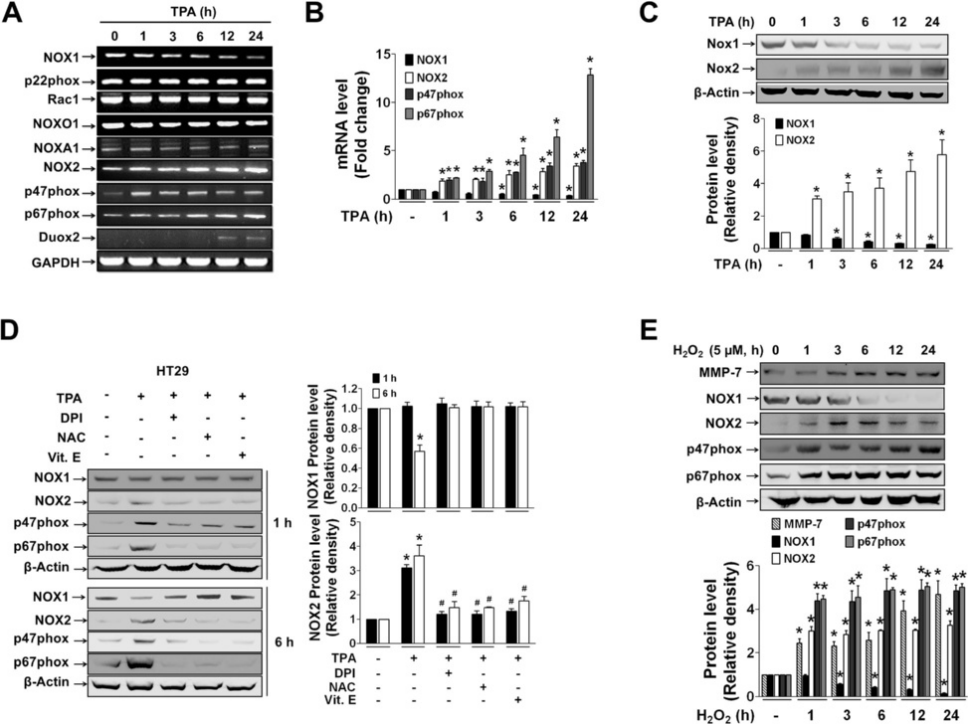
Expression of NOX2 and its regulator subunits, p47phox and p67-phox, is regulated by NOX2-derived ROS in TPA-treated HT29 cells. **a-b** HT29 cells treated with TPA (12 ng/ml) for the indicated time periods were analyzed for gene expression by conventional PCR (**a**), qRT-PCR (**b**), and Western blotting (**c**). **d** Protein extracts from HT29 cells treated with DPI, NAC, and Vit. E in the presence of TPA were immunoblotted with anti-NOX2, p47phox, and p67-phox antibodies. β-Actin was used for equal loading. **e** HT29 cells were stimulated with H_2_O_2_ (5 μM) for the indicated time, and Western blotting was performed for detection of MMP-7, NOX2, p47phox, and p67-phox. Bands are representative of three independent experiments, and bar graphs indicate the mean ± SEM of relative densities of the gene products. *P < 0.05 compared to vehicle-treated control group. ^#^ *P* < 0.05 compared to TPA-treated group

HT29 cells expressed all components required for activation of NADPH oxidase, the catalytic core subunits p22phox, NOX1, and NOX2, and the regulatory subunits NOXO1, NOXA1, p47phox, p67phox, and Rac1 (Fig. 3a). Treatment of HT29 cells with TPA suppressed expression of NOX1 with significant changes starting at 3 h (Fig. 3b and c). In contrast to NOX1, TPA increased mRNA expression levels of NOX2, p47phox, and p67phox in a time-dependent manner, and the degree of induction was strongest for p67phox (Fig. 3b and c). The TPA-induced increase in NOX2 accompanying with reduction of NOX1 protein expression was also observed in Caco2 cells (Additional file 2: Figure S2). The increased expression of NOX2 and its regulators was significantly suppressed by DPI as well as antioxidants (Vit. C and Vit. E) starting from 1 h until 6 h, whereas ROS suppressed NOX1 expression only at 6 h (Fig. 3d). These results indicate that NOX2-derived ROS increase expression of NOX2 and its regulators, p47phox and p67phox, as well as reduce NOX1 expression. To further confirm, cells directly exposed to exogenous ROS were examined for induction of these same genes. Application of exogenous H_2_O_2_ (5 μM) resulted in increased expression of NOX2, p67phox, and p47phox as well as MMP-7 in a time-dependent manner (Fig. 3e). In contrast, reduced NOX1 expression was observed from 3 h after H_2_O_2_ treatment.

**Fig. 4:**
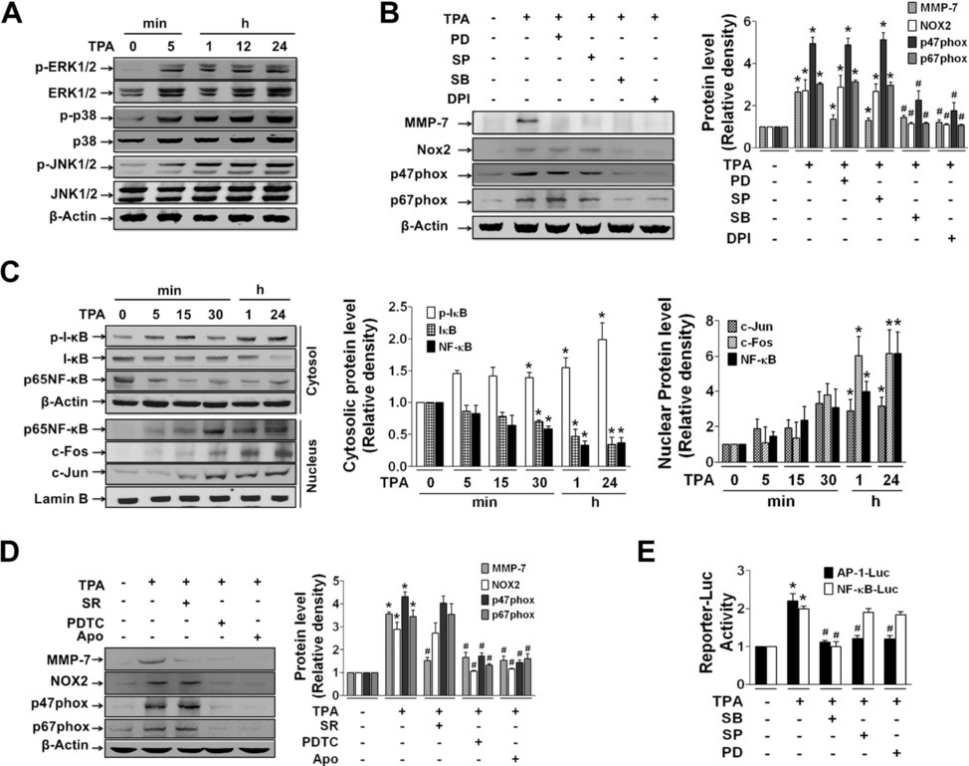
Expression of NOX2 and its regulators is mediated through p38 and NF-κB, whereas MMP-7 expression is mediated through MAPKs, AP-1, and NF-κB. Protein extracts from cells treated with TPA (12 ng/ml) alone or TPA with various inhibitors were examined for phosphorylation of MAPKs (**a**), expression of NOX2, its regulators, and MMP-7 (**b** and **d**), and nuclear translocation of the transcription factors NF-κB and AP-1 (**c**). PD, SP, SB, and SR represent 10 μM PD98059, SP600125, SB203580, and SR11302, respectively. The bar graphs indicate the mean ± SEM of relative densities of protein expression. **e** The luciferase reporter plasmid containing human NF-κB or AP-1 response element was transiently transfected, and reporter activity was assessed following incubation of transfected cells with TPA for 3 h. Various inhibitors were pretreated 1 h before TPA treatment. Values are the mean ± SEM from three independent experiments. *P < 0.05 compared to vehicle-treated control group. ^#^ *P* < 0.05 compared to TPA-treated group

We next investigated the involvement of MAPKs as downstream signaling molecules for gene induction. TPA increased phosphorylation of MAPKs, ERK, p38, and JNK starting at 5 min continuously up to 24 h (Fig. 4a). PD98059 and SP600125 (ERK and JNK inhibitors, respectively) did not suppress TPA-induced expression of NOX2 or its regulators, whereas SB203580 (p38 inhibitor) did show suppressive effects similar to those of DPI (Fig. 4b). In contrast, MMP-7 gene expression was suppressed by PD98059, SP600125, SB203580, and DPI (Fig. 4b). In addition, TPA activated the transcription factors NF-κB, c-Fos and c-Jun in HT29 cells (Fig. 4c), although their involvement in gene regulation varied. TPA-induced MMP-7 expression was suppressed by SR11302 and PDTC (AP-1 and NF-κB inhibitors, respectively), whereas NOX2, p47phox, and p67phox expression was inhibited by PDTC but not SR11302 (Fig. 4d). Further, in reporter gene analysis using a reporter plasmid containing human NF-κB (NF-κB-Luc) or AP-1 (AP-1-Luc) response element, TPA-enhanced AP-1-Luc reporter activity was significantly suppressed by SB203580, PD98059, and SP600125, whereas TPA-enhanced NF-κB-Luc reporter activity was inhibited only by SB203580 (Fig. 4e).

### AMPK activity and expression of NOX2 and MMP-7 are inversely regulated by ROS production in HT29 colon cancer cells

**Fig. 5:**
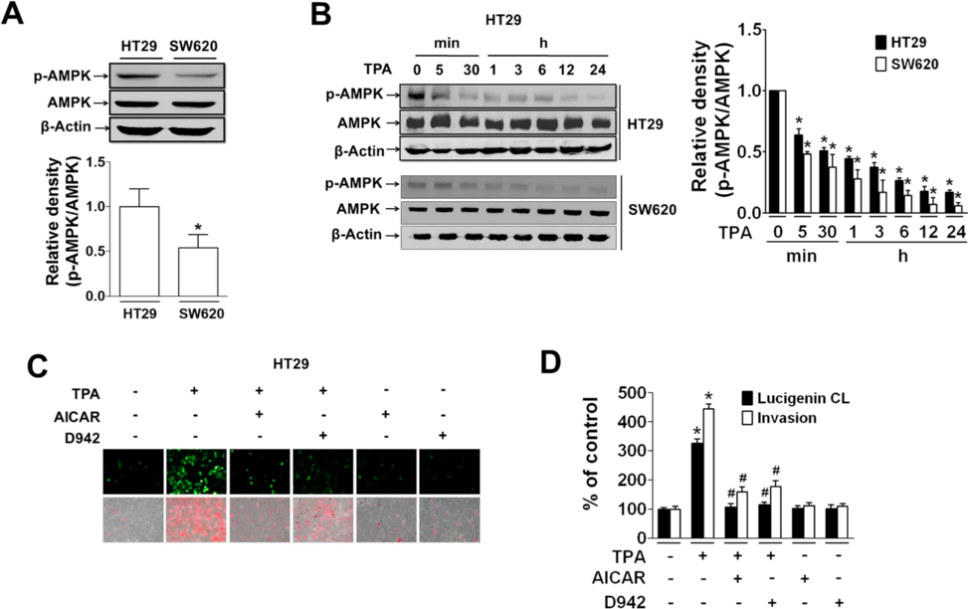
Reduced AMPK phosphorylation corresponds to increased ROS production and invasion of TPA-treated HT29 cells, which is reversed by AMPK activators. **a** Basal AMPK phosphorylation was compared between HT29 and SW620 cells. The bar graph represents the mean ± SEM of phospho-AMPK/AMPK from three independent experiments. *P < 0.05 compared to HT29 cells. **b** Protein extracts from TPA-treated HT29 cells were examined for phospho-AMPK by Western blotting. **c** HT29 cells were treated with AMPK activators, AICAR (250 μM) and D942 (10 μM), in the presence or absence of TPA for 1 h. Cells were then examined for ROS production (upper panel) by DCF fluorescence microscopy and invasion (lower panel). **d** Superoxide anion production was measured by lucigenin chemiluminescence assay, and invasion was quantitated by counting cells on the bottom of the insert. The bar graph represents the mean ± SEM from three independent experiments. *P < 0.05 compared to vehicle-treated control group. ^#^ *P* < 0.05 compared to TPA-treated cells

We also investigated AMPK activity in response to ROS during colon cancer cell invasion. Under serum-starved conditions, AMPK phosphorylation was higher in HT29 cells than in SW620 cells (Fig. 5a). TPA treatment inhibited AMPK phosphorylation in HT-29 cells (Fig. 5b) starting from 5 min after treatment, which is in line with increased ROS production (Fig. 2a and g) and subsequent invasion. In SW620 cells, reduction of AMPK phosphorylation by TPA occurred in a similar pattern compared to that in HT29 cells. In addition, pretreatment with AICAR and D942 (AMPK activators) significantly blocked TPA-induced ROS production and cell invasion (Fig. 5c and d). On the other hand, treatment with AICAR or D942 alone without TPA did not cause any change in ROS production or cell invasion.

**Fig. 6:**
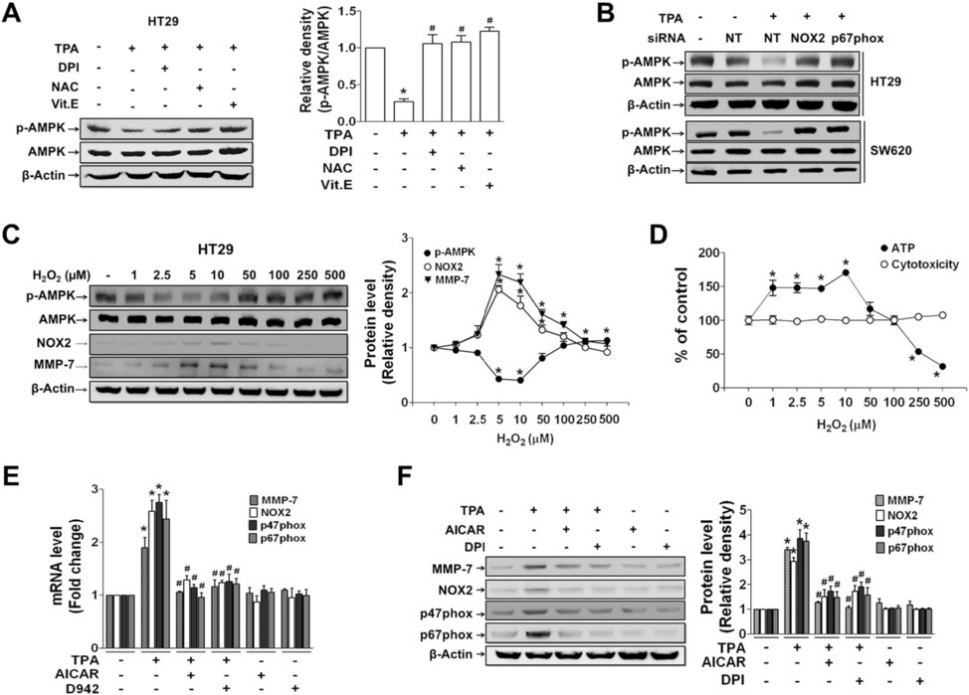
TPA-induced reduction of AMPK phosphorylation is dependent on NOX2-derived ROS production. **a** Protein extracts from HT29 cells pretreated with DPI (10 μM), NAC (10 μM), and Vit. E (50 μg/mL) for 1 h prior to treatment with TPA (1 h) were examined for phospho-AMPK/AMPK levels. **b** HT29 and SW620 cells transfected with siRNA specific to NT, NOX2, or p67phox were treated with TPA and analyzed for expression of phospho-AMPK. **c** HT29 cells were treated with exogenous H_2_O_2_ at the indicated concentrations for 1 h, and cell extracts were examined for levels of phospho-AMPK/AMPK, NOX1, NOX2, and MMP-7. **d** HT29 cells treated with H_2_O_2_ were analyzed for cellular ATP level using a Mitochondrial ToxGlo™ assay kit. Cytotoxicity was measured at the same time using the kit. (**e, f**) HT29 cells treated with AICAR or D942 in the presence or absence of TPA were analyzed for mRNA (**e**) or protein (**f**) expression. The bar and line graphs show the mean ± SEM from three independent experiments. *p < 0.05 compared to control, ^#^ *p* < 0.05 compared to TPA-treated group

We next investigated any link between reduced AMPK phosphorylation and NOX2-derived ROS production. TPA-induced reduction of AMPK phosphorylation was blocked by DPI and antioxidants (NAC and Vit. E) (Fig. 6a). Reduction of AMPK phosphorylation was also blocked by pretreatment with siRNAs specific to NOX2 and p67phox, which was commonly observed in both HT29 and SW620 cells (Fig. 6b). Based on these results, AMPK phosphorylation was apparently inhibited by TPA-induced ROS production, which contradicts previous reports that ROS increases phosphorylation of AMPK [48, 49]. To determine whether or not the ROS level can explain this discrepancy, we investigated AMPK activity in response to different concentrations of ROS. HT29 cells exposed to different concentrations of exogenous H_2_O_2_ responded differently, showing a U-shape response curve for AMPK phosphorylation. Specifically, AMPK phosphorylation decreased in the presence of 10 μM H_2_O_2_ and increased at concentrations over 10 μM H_2_O_2_ (Fig. 6c). On the contrary, NOX2 and MMP-7 expression showed opposite patterns (Fig. 6c). Treatment with 1 to 500 μM H_2_O_2_ did not affect cell viability (Fig. 6d). Further, pretreatment of cells with AMPK activators suppressed TPA-induced expression of NOX2, p47phox, p67phox, and MMP-7 (Fig. 6e and f), and the effect was similar to that of DPI (Fig. 6f). However, treatment of cells with AMPK activators in the absence of TPA did not affect mRNA (Fig. 6e) or protein (Fig. 6f) expression of NOX2 and MMP-7, which is similar to the case of basal ROS production and invasion in HT29 cells (Fig. 5c and d).

## Discussion

MMP-7 is unique in that it is predominantly expressed in epithelial cells as opposed to other MMPs, which are mostly expressed in the stroma [50, 51]. Overexpression of MMP-7 in human squamous cell carcinomas, in particular colon cancer cells, shows a strong positive correlation with metastatic potential of cancer cells [52–54]. In the current study, we observed that MMP-7 was differentially expressed in colon cancer cell lines, HT29, Caco2, SW620, and HCT 116, having different invasive potentials. The TPA-induced transition from less (HT29 and Caco2 cells) to highly metastatic (SW620 and HCT116 cells) was associated with enhanced MMP-7 expression, which was dependent on the molecular switch from NOX1 to NOX2.

MMP gene expression is not constitutive but is rather regulated by various cytokines via activation of intracellular signaling pathways [55]. In our current study, complete blockage of TPA-induced invasion of both HT29 and SW620 cells was observed in the case of either MMP-7 silencing or pretreatment with DPI and Apo (NADPH oxidase inhibitors), indicating a regulatory role for NADPH oxidase in MMP-7 induction as well as cancer cell invasion. It has been reported that MMP-7 expression increases in response to exogenous H_2_O_2_ [15]. In this study, we clearly observed that the source of ROS governing MMP-7 expression in colon cancer cells was NADPH oxidase. In addition, the current results of NADPH oxidase activity measurement showing that NOX1 siRNA had no effect on TPA-induced ROS generation demonstrate that TPA-induced ROS was independent of NOX1 despite high NOX1 levels in HT29 cells. A previous study by Sadok *et al.*, [56] showed that NOX1 silencing using siRNA decreases superoxide production in HT29-D4 cells. This contradictory result could be attributed to a difference in the type of stimulus given to cells, because in the previous study, cells were induced to be in migratory phase by being placed on collagen-I which activates integrin signaling. However, further study is required for the underlying mechanism to be solved. Our current results also demonstrate that treatment of HT29 cells with TPA reduced NOX1 expression to a level similar to that in untreated SW620 cells. These results are somewhat in line with previous reports that NOX1 is constitutively expressed in the colon epithelium to a similar degree as in differentiated colon cancer cells [57], and increased NOX1 expression suppresses proliferation of cancer cells, driving them into a differentiated cancer state [58]. An important role of NOX1 in tumorigenesis has been suggested based on several findings. Induction of NOX1 mRNA transcription has been observed in non-cancerous cells such as smooth-muscle cells, and fibroblasts by stimulation with platelet-derived growth factor or epidermal growth factor [59, 60]. It is also reported that K-Ras mutation correlates with increased NOX1 mRNA expression and colon tumor phenotype [61]. However, based on human tumor tissue array findings of diminished NOX1 expression at a more advanced tumor stage [58], it is suggested that tumor-promoting action of NOX1 seems to be an early event [62]. Our current results showing that NOX1 expression is diminished as colon cancer cells undergo invasive phenotype change also support such suggestion.

In contrast to NOX1, NOX2 expression was elevated and accompanied by enhanced MMP-7 expression in TPA-treated HT29 cells, which was similar to that in basal SW620 cells. In addition, NOX2 siRNA inhibited basal NADPH oxidase activity, TPA-induced ROS production, MMP-7 expression, and cell invasion, indicating that NOX2 plays a critical role in induction of MMP-7 expression in invasive cancer cells. These results further indicate that switch from NOX1 to NOX2 induces colon cancer cells to be highly invasive. In support of this, ROS have been reported to play signaling roles in normal cells such as cardiomyocytes and keratinocytes for pro-survival [63] or MMP-9 induction [64]. Likewise, the results of the current study demonstrate that MMP-7 expression was accompanied by activation of MAPKs as well as the transcription factors NF-κB and AP-1. At the same time, NOX2-activated ROS constituted an autoregulatory loop for expression of NOX2 and its regulators, p47phox and p67phox in TPA-treated colon cancer cells. This is similar to the previous finding that expression of phagocytic NADPH oxidase subunits is up-regulated by stimulus-induced ROS in a positive feedback mechanism [65].

AMPK activation in colon cancer cells has been reported to suppress the expression of genes associated with invasion and metastasis, including integrin β1 and cyclooxygenase-2 [37, 38]. In the current study, we observed for the first time that AMPK activity was inversely linked to MMP-7 expression in colon cancer cells. At a basal level, low MMP-7 expression and high AMPK phosphorylation were observed in less invasive HT29 cells, whereas low AMPK phosphorylation and high MMP-7 expression were observed in SW620 cells. Such an inverse relationship between AMPK activity and MMP-7 expression was further confirmed based on our observations that TPA-induced MMP-7 expression was accompanied by reduced AMPK phosphorylation and that AMPK activator blocked TPA-induced MMP-7 expression. In addition, a low concentration (5 μM) of H_2_O_2_ suppressed AMPK phosphorylation, which was prevented by DPI or antioxidants as well as siRNAs specific to NOX2 and p67phox. This result indicates that AMPK phosphorylation was suppressed by NOX2-activated ROS production in TPA-treated HT29 cells. Gene expression in response to NOX2-activated ROS production in HT29 cells was very similar to that in 5 μM H_2_O_2_-treated cells. Further, we observed for the first time that AMPK phosphorylation was differentially regulated according to ROS concentration, showing a U-shape dose–response curve associated with ATP production in HT29 cells. Low concentration of H_2_O_2_ up to 10 μM resulted in increased ATP production and a corresponding decrease in AMPK phosphorylation, which was reversed at above 10 μM H_2_O_2_. Our current study, which used a full range of H_2_O_2_ concentrations, actually supports previous findings showing that ROS activates AMPK [34, 66, 67]. However, in contrast to previous reports of a direct relationship between AMPK activation and mitochondrial ROS production in cancer cells [32], our results suggest that plasma membrane NOX2-derived ROS production deactivates AMPK. Despite our results that AMPK phosphorylation and ATP production are exactly matched in response to H_2_O_2_ treatment, the underlying mechanism must be elucidated.

AMPK phosphorylation was inversely correlated with the expression level of NOX2, indicating that there is a feedback loop between NOX and AMPK. However, AMPK activators alone in the absence of TPA did not suppress basal ROS production or NOX2 gene expression, suggesting that activation of NOX2 accompanied by ROS generation is a prerequisite of AMPK suppression. Further, our results suggest that deactivated AMPK did not exert any inhibitory effect on stimulus-induced NOX2 expression and thus MMP-7 gene induction over time.

## Conclusions

**Fig. 7:**
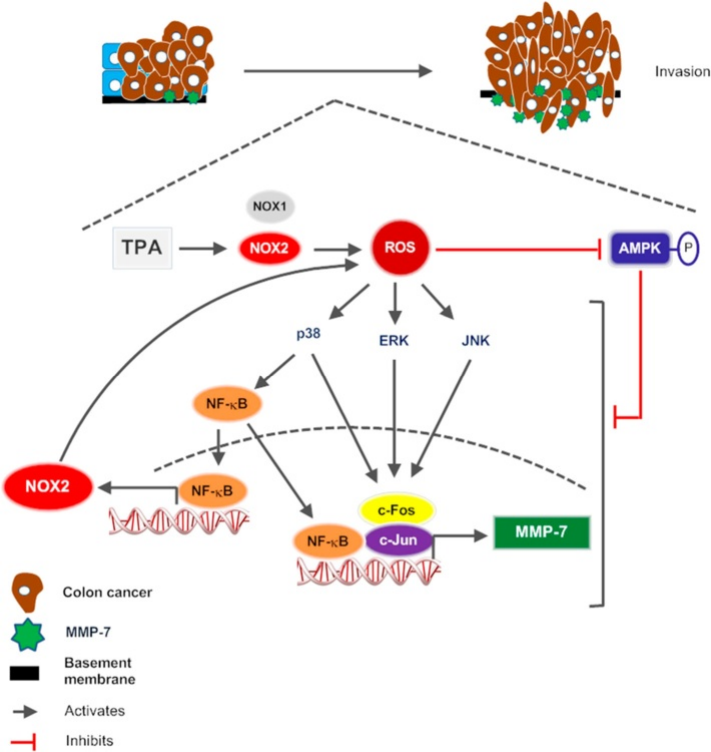
Molecular switch from NOX1 to NOX2 induces invasive phenotype change in colon cancer cells. NOX2-derived ROS activates MAPKs, p38, ERK, and JNK with deactivation of AMPK. P38 activates NF-κB, which induces NOX2, p47phox, and p67phox. At the same time, p38, ERK, and JNK activate AP-1, which up-regulates MMP-7 gene expression in cooperation with NF-κB

MMP-7 overexpression drives colon cancer cells into an highly invasive phenotype through molecular switch from NOX1 to NOX2. NOX2-derived ROS production reduces NOX1 gene expression and enhances expression of NOX2 and MMP-7 by deactivating AMPK, which otherwise exerts an inhibitory effect against stimulus-induced autoregulation of ROS and NOX2 gene expression. The study provides a comprehensive molecular mechanism of the invasive phenotype in colon cancer cells (Fig. 7).

## Methods

### Materials

All reagents were purchased from Sigma-Aldrich (St. Louis, MO, USA), if not specified. RPMI1640, MEM, fetal bovine serum (FBS), penicillin/streptomycin, and trizol reagent were purchased from Invitrogen Life Technologies (Carlsbad, CA, USA). Antibody of Lamin B, I-κB-α rabbit polyclonal antibody, β-actin mouse monoclonal antibody, NF-κB p65 rabbit polyclonal antibody, phospho-ERK antibody, MMP-7 goat polyclonal antibody, p67phox goat polyclonal antibody, NOX1 rabbit polyclonal antibody, NOX2 goat polyclonal antibody, celecoxib were obtained from Santa Cruz Biotechnology (Santa Cruz, CA, USA). Phospho-p38 MAPK antibody, ERK antibody, p38 MAPK antibody, phospho-AMPK-α, AMPK-α, phospho-JNK, JNK, C-Jun, phospho I-κB, and PD98059 were purchased from Cell Signaling Technology, Inc. (Boston, MA, USA). Matrigel was obtained from BD Biosciences (Bedford, MA, USA). Trypsin/EDTA was purchased from Clonetics, Inc. (Walkersville, MD, USA). SR11302 was purchased from Tocris Bioscience (Tocris House, Bristol, BS110QL, UK). D942 was purchased from Calbiochem (10394, Pacific Center Ct, CA, USA).

### Cell culture

Human colorectal cancer cells HT29 and SW620 were obtained from the American Type Culture Collection (Manassas, VA, USA). HT29 cells and SW620 cells were cultured in RPMI 1640 and DMEM high glucose media, respectively, containing 10 % FBS, 100 IU/ml of penicillin, and 100 μg/ml of streptomycin. Cells were maintained at 37 °C in 5 % CO_2_. After reaching 70 % confluence, cells were sub-cultured by splitting at 1:3 ratios.

### Quantitative real-time polymerase chain reaction (qRT-PCR)

Total RNA was isolated using Trizol reagent following the manufacturer’s instructions. The extracted RNA was reverse-transcribed to cDNA using a GoScript Reverse Transcription system (#A5001, Promega Corporation, WI, USA). cDNA was amplified in the presence of 0.5 U of Taq DNA polymerase (Takara, Japan) using specific primers on Corbett Rotor-Gene (Corbett Life Science). Quantitative analysis of mRNA was done using a QuantiTect SYBR Green PCR kit (Qiagen). Primers for MMP-2, MMP-9, TIMP-1, TIMP-2, and GAPDH were supplied by Qiagen, and the other primer sequences used were MMP-7 (sense 5’-GGAGATGCTCACTTCGATGA-3’ and antisense 5’-ATACCCAAAGAATGGCCAAG -3’), NOX1 (sense 5′-GTTTTACCGCTCCCAGCAGA −3′ and antisense 5′-GGATGCCATTCCAGGAGAGA-3′), NOX2 (sense 5′-CCTAAGATAGCGGTTGATGG-3′ and antisense 5′-GACTTGAGAATGGATGCGAA -3′), p47phox (sense 5′-GCTGGTGGGTCATCAGGAAA-3′ and antisense 5′-GCCCTGACTTTTGCAGGTAC −3′), and p67phox (sense 5′-CCTGCAACTACCTTGAACCA −3′ and antisense 5′- GGACTGCGGAGAGCTTTCC-3′).

### Matrigel invasion assay

Matrigel invasion assay was performed as described previously by Park *et al.* [46]. Briefly, inner and outer parts of the Transwell insert (BD Falcon, Franklin Lakes, USA) were coated with Matrigel (0.5 mg/ml) and collagen (1 mg/ml) respectively. TPA was added to all wells except the control well, after which 100 μl of cell suspension (5x10^5^ cells/ml) in the presence or absence of inhibitors and antioxidants in serum-free media was added to the inner part of the transwell. After 24 h, invaded cells were fixed and stained with methanol and H&E separately. The number of invaded cells per field was captured using a microscope fitted with a camera at 200x magnification.

### Subcellular fractionation and Western blot analysis

For subcellular fractionation, HT29 cells cultured in a 100-mm dish were serum-starved overnight and stimulated with TPA (0 to 60 min). Cells were harvested with 100 μl of subcellular fractionation buffer (SF) consisting of 250 mM sucrose, 20 mM HEPES (pH 7.4), 10 mM KCl, 1.5 mM MgCl2, 1 mM EDTA, 1 mM EGTA, 1 mM Dithiothreitol, 1x protease, and phosphatase inhibitor cocktail. Cells were passed through a 30 G needle 30–35 times on ice. The cell suspension was centrifuged at 700 g for 10 min at 4 °C. The supernatant was collected in a glass tube and centrifuged at 100,000 rpm (Sorvall RC M120 EX ultracentrifuge) for 1 h at 4 °C. After collecting supernatant containing the cytosolic fraction, the pellet was suspended in 60 μl of SF. The suspension was sonicated for 30 s four times at the highest setting with a 30-s break in each cycle. The suspension was centrifuged again at 100,000 rpm for 1 h at 4 °C, and supernatant containing the membrane fraction was collected.

Cytoplasmic and nuclear proteins were extracted using a NE-PER nuclear and cytoplasmic extraction reagent kit (#78833, Thermo Scientific, Rockford, USA) according to the manufacturer’s instructions. For isolation of total proteins, cells were lysed in radioimmunoprecipitation assay buffer (RIPA) containing protease and phosphatase inhibitor cocktail (Thermo Scientific). Cells were centrifuged at 12,000 rpm for 10 min, and supernatant containing soluble proteins was collected.

Protein concentration was determined using a BCA protein assay kit (Thermo Scientific). Equal quantities of proteins were resolved by 10-14 % SDS-PAGE, transferred onto a nitrocellulose membrane, and blocked using 5 % skim milk in TBS-Tween 20 (TBST) for 1 h. In the case of phospho-protein, membranes were blocked in 5 % bovine serum albumin in TBST for 1 h. Membranes were incubated with specific primary antibodies overnight at 4 °C, followed by incubation with horseradish peroxidase-conjugated secondary antibody for 1 h at room temperature. The immunoreactive proteins were detected using an enhanced chemiluminescent reagent (Thermo Scientific) system on a luminescent image analyzer, LAS-4000 mini (Fujifilm, Tokyo, Japan). In each step after blocking, membranes were washed with TBST.

### Transfection of colon cancer cells with siRNA

HT29 and SW620 cells were transiently transfected using siRNAs (NOX1, NOX2, p67phox, and MMP-7) in opti-MEM I medium containing Dharmacon reagent (# T-2004-01, Thermo Scientific) as previously described by Regmi *et al.* [68]. The transfected cells were then subjected to invasion assay, ROS measurement, and Western blot analysis.

### Lucigenin Chemiluminescence assay

Superoxide anion production was measured by lucigenin chemiluminescence assay as described by Regmi *et al.* [68] with some modifications. Cells were seeded in 96-well white opaque plates (1×10^5^ cells/well). After overnight incubation, cells were pretreated with drugs in serum-free media prior to treatment with or without TPA (12 ng/ml) for the indicated time. Chemiluminescence was then measured using lucigenin (400 μM) with a Fluostar Optima microplate reader.

### Intracellular ROS measurement

Cellular ROS was measured using a cell-permeable fluoregenic probe, 2’,7’-dichlorofluorescein diacetate (DCF-DA). Overnight serum-starved HT-29 cells (1×10^5^ cells/cm^2^) were treated with or without TPA for the specific time points. After washing with PBS, cells were incubated with 10 μM DCF-DA at 37 °C for 30 min. The cells were washed again with PBS, and images were captured using a digital camera (TE2000-U, Nikon, Japan) with a blue filter (B-2E/C, FITC) at 200x magnification.

### mRNA copy number determination

The number of transcripts of NOX1 and NOX2 was determined as previously described methods [68, 69]. Briefly, human NOX1 and Nox2 cDNAs (Invitrogen) were cloned into pcDNA5/FRT/TO vector (Invitrogen). The following equation was used to calculate the copy number:

$$ \mathrm{D}\mathrm{N}\mathrm{A}\ \left(\mathrm{copies}\right) = \left[6.02 \times 1023\ \left(\mathrm{copies}/\mathrm{mol}\mathrm{e}\right) \times \mathrm{D}\mathrm{N}\mathrm{A}\ \mathrm{concentration}\ \left(\mathrm{g}\right)\right]\ /\ \left[\mathrm{D}\mathrm{N}\mathrm{A}\ \mathrm{length}\ \left(\mathrm{bp}\right) \times 660\ \left(\mathrm{g}/\mathrm{mol}/\mathrm{bp}\right)\right]. $$

To generate standard curve of NOX1 and NOX2, each plasmid were serially diluted 10 folds ranging from 10^1^ to 10^5^ copy numbers. cDNA synthesized from isolated total RNA of cells in the absence or presence of TPA for 24 h were subjected to real-time PCR using QuantiTect SYBER Green PCR kit (Qiagen) with NOX1 and Nox2 primer sequences. Copy number of NOX1 and Nox2 in HT29, Caco-2, SW620, HCT116 was calculated from the standard curve.

### NADPH Oxidase activity assay

The NADPH oxidase activity assay was performed by modifying the method described by [70]. Briefly, cells were transfected with siRNAs of non-specific, NOX1 and NOX2. The cells were harvested in Krebs-HEPES buffer (pH 7.4) containing protease and phosphatase inhibitor cocktail (Thermo Scientific), homogenized with Dounce homogenizer, and centrifuged at 10,000 g for 15 min. After determining protein concentration using a BCA protein assay kit (Thermo Scientific), equal amount of proteins was transferred to the 96 well (white plate) with 10 μM lucigenin prepared in the same buffer and incubated at 37 °C for 10 min. Then, 100 μM NADPH was added to each wells with or without TPA. After 5 min incubation at 37 °C, the chemiluminescence was measured with a Fluostar Optima microplate reader.

### Measurement of ATP

Mitochondrial function was assessed using Mitochondrial ToxGlo™ Assay (#G8000, Promega). Briefly, HT-29 cells (10,000 cells/well) were seeded in a 96-well opaque white-walled flat bottom plate (Falcon). After 24 h of incubation at 37 °C, cells were washed with PBS, and serum-free galactose-containing media (glucose-free) was added to each well. Cells were treated with different concentrations of H_2_O_2_ (1 to 500 μM) or TPA (12 ng/ml) for 1 h. Cytotoxicity was first assessed using fluorogenic peptide substrate (bis-AAF-R110). Fluorescence was measured using a Fluostar optima microplate reader (BMG Labtech GmbH, Offenburg, Germany) with excitation at 485 nm and emission at 520 nm. Next, cells were lysed by addition of ATP detection reagent, and luminescence was measured using a Fluostar optima microplate reader following the manufacturer’s instructions from the Mitochondrial ToxGlo™ assay kit. The amount of ATP is directly proportional to the luminescence signal.

### Reporter plasmid transfection and Luciferase activity measurement

Transactivation of NF-κB and AP-1 was studied using the dual Luciferase reporter assay system (Promega, Madison, WI) following the manufacturer’s instructions. Briefly, HT29 cells were seeded in a 24-well plate at a density of 7×10^4^ cells/well in antibiotic-free media containing 10 % FBS. The next day, cells were co-transfected with NF-κB (Affymetrix) or AP-1 (Panomics) reporter vectors along with control vector (pRL-TK) using Lipofectamine 2000. The transfection media was replaced with RPMI 1640 containing 10 % FBS after 18 h, and the cells were further allowed to grow for another 24 h. Then, the transfected cells were treated with MAPK inhibitors for 1 h prior to treatment with TPA for another 3 h. The lysates were used for the assay.

### Statistical analysis

All data are the mean of three independent experiments. Error bar represents SEM Statistical significance was determined using Student’s *t*-test or one-way ANOVA followed by the Student-Newman-Keuls comparison method for calculation of differences between groups (GraphPad Prism 5.0 software, San Diego, CA, USA). Values of *P* < 0.05 were considered statistically significant.

## Additional files

Additional file 1: Figure S1. Differentially expressed MMP-7 in colon cancer cell lines having different invasive potentials was increased by TPA. **A** The mRNA level of MMP-7 and MMP-9 in HT29, Caco2, SW620, and HCT116 cells was measured by qRT-PCR. **P* < 0.05 compared to HT29 or Caco2 cells. **B** MMP-7 protein expressions in TPA-treated HT29, Caco2, SW620, and HCT116 cells were analyzed by Western blot method. The bar graphs indicate the mean ± SEM of relative densities of MMP-7 protein expression. **P* < 0.05 compared to vehicle-treated control group. Additional file 2: Figure S2. TPA induces decrease in NOX1 and increase in NOX2 in Caco2 cells. Proteins were extracted from Caco2 cells treated with TPA for the indicated time period, and analyzed by Western blot. The bar graphs indicate the mean ± SEM of relative densities of NOX1 and NOX2 protein expressions. **P* < 0.05 compared to vehicle-treated control group.

## Abbreviations

ATCC: American type culture collection
AMPK: AMP-activated protein kinase
AICAR: 5-amino-1-β-D-ribofuranosyl-imidazole-4-carboxamide
ANT: Antimycin A
Cele: Celecoxib
DCF-DA: 2’,7’-dichlorofluorescein diacetate
DPI: Diphenyleneiodonium
D942: 5-(3-(4-(2-(4-Fluorophenyl)ethoxy)phenyl)propyl)furan-2-carboxylic acid
FBS: Fetal bovine serum
GF: GF109203X
MAPK: Mitogen-activated protein kinase
MMP: Matrix metalloproteinase
NAC: N-acetylcysteine
NOX: NADPH oxidase
RIPA: Radioimmunoprecipitation assay
ROS: Reactive oxygen species
RLU: Relative luminescence unit
qRT-PCR: Quantitative real time-polymerase chain reaction
SF: Subcellular fractionation
TPA: 12-O-tetradecanoylphorbol-13-acetate
TBST: Tris-buffered saline (TBS)-Tween 20
Vit. E: Vitamin E
