# Supplementary figures and images for: NOX1 to NOX2 switch deactivates AMPK and induces invasive phenotype in colon cancer cells through overexpression of MMP-7
Source: Mol Cancer. 2015 Jun 27;14:123. doi: 10.1186/s12943-015-0379-0 (PMC4482031; doi:10.1186/s12943-015-0379-0)

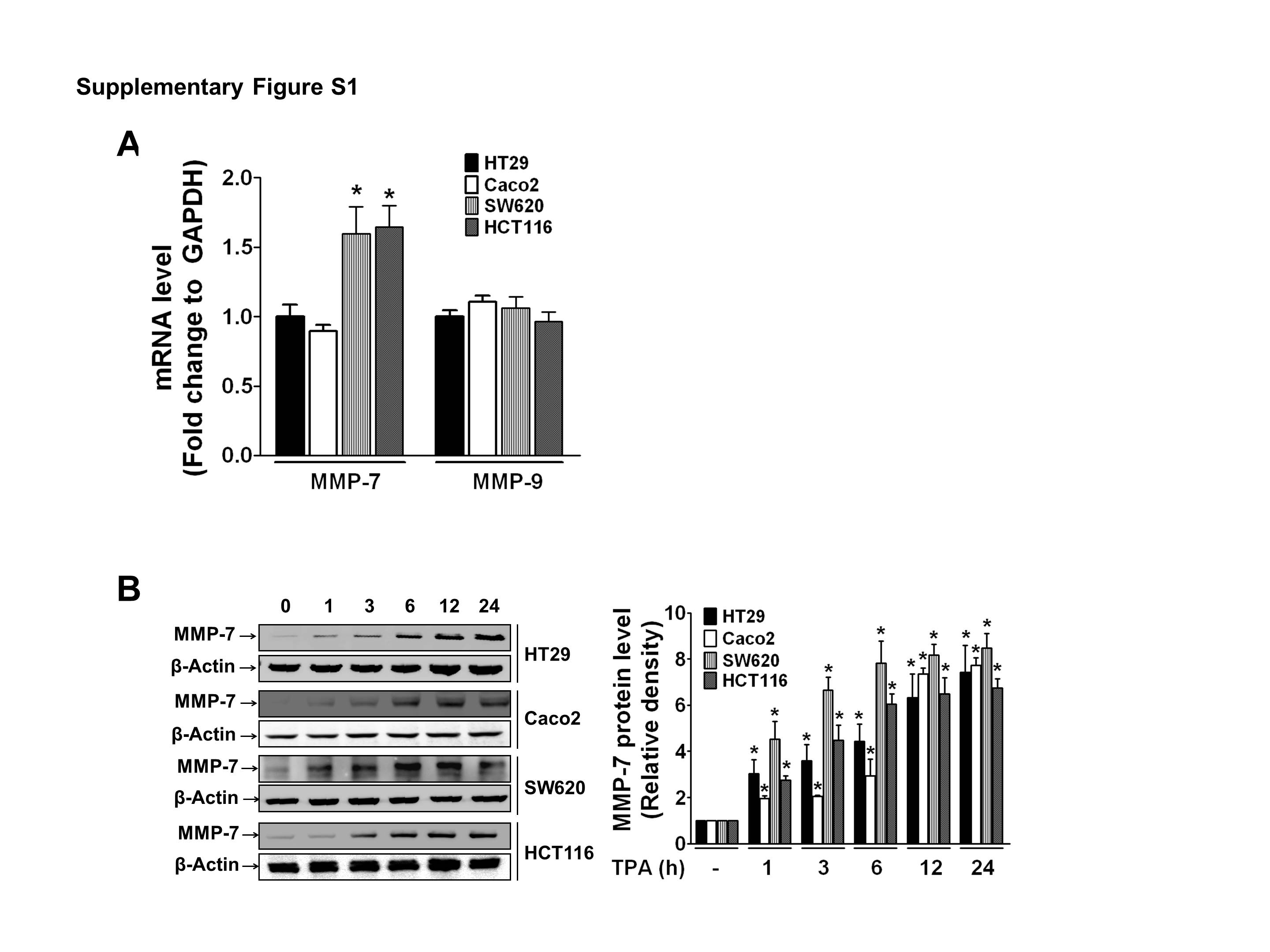

Supplement: Additional file 1: Figure S1. — Differentially expressed MMP-7 in colon cancer cell lines having different invasive potentials was increased by TPA. A The mRNA level of MMP-7 and MMP-9 in HT29, Caco2, SW620, and HCT116 cells was measured by qRT-PCR. *P < 0.05 compared to HT29 or Caco2 cells. B MMP-7 protein expressions in TPA-treated HT29, Caco2, SW620, and HCT116 cells were analyzed by Western blot method. The bar graphs indicate the mean ± SEM of relative densities of MMP-7 protein expression. *P < 0.05 compared to vehicle-treated control group. [file 12943_2015_379_MOESM1_ESM.jpeg]

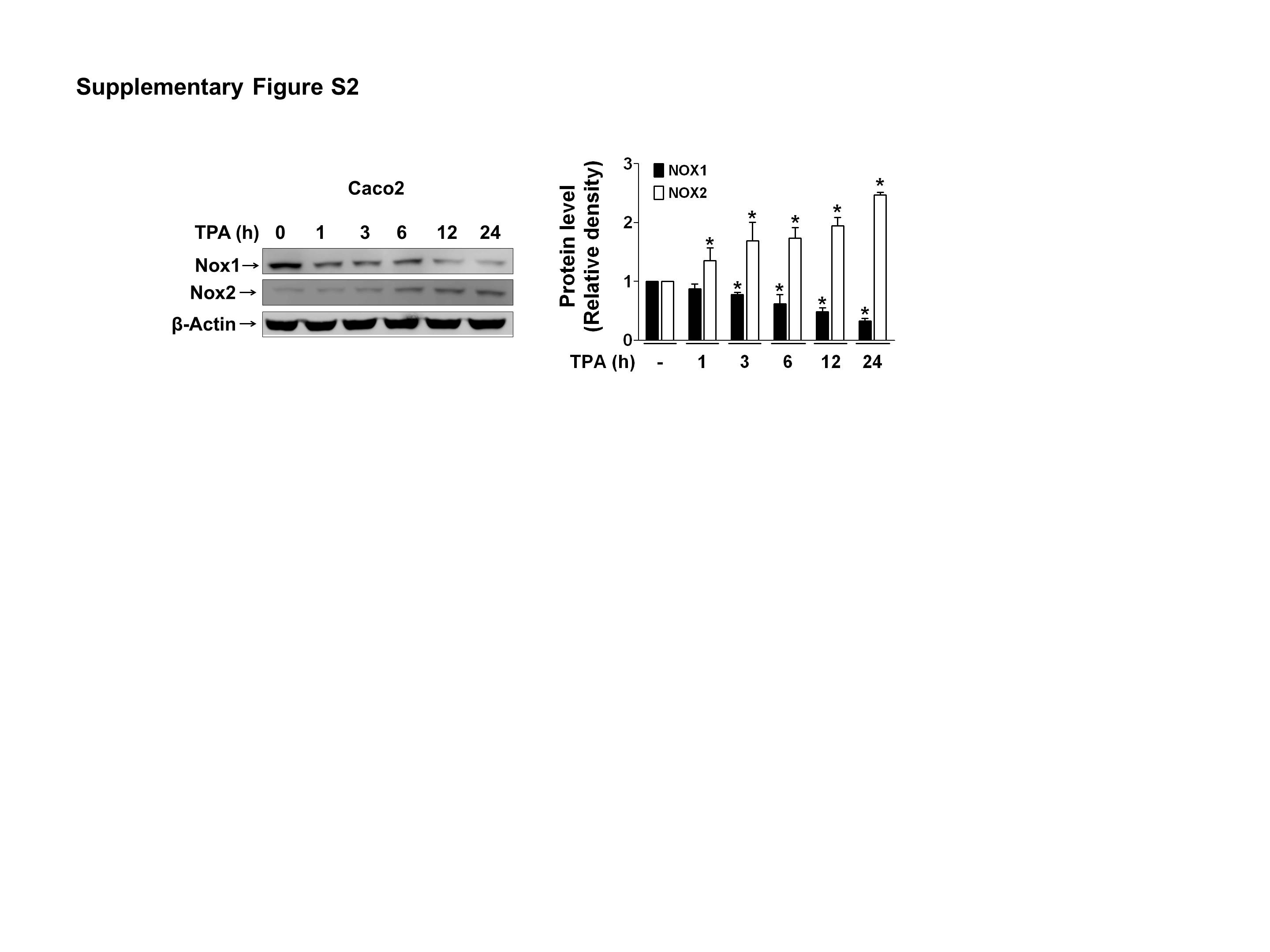

Supplement: Additional file 2: Figure S2. — TPA induces decrease in NOX1 and increase in NOX2 in Caco2 cells. Proteins were extracted from Caco2 cells treated with TPA for the indicated time period, and analyzed by Western blot. The bar graphs indicate the mean ± SEM of relative densities of NOX1 and NOX2 protein expressions. *P < 0.05 compared to vehicle-treated control group. [file 12943_2015_379_MOESM2_ESM.jpeg]
